# Theoretical Study on Transverse Mode Instability in Raman Fiber Amplifiers Considering Mode Excitation

**DOI:** 10.3390/mi15101237

**Published:** 2024-10-07

**Authors:** Shanmin Huang, Xiulu Hao, Haobo Li, Chenchen Fan, Xiao Chen, Tianfu Yao, Liangjin Huang, Pu Zhou

**Affiliations:** 1 College of Advanced Interdisciplinary Studies, National University of Defense Technology, Changsha 410073, China; 2 Nanhu Laser Laboratory, National University of Defense Technology, Changsha 410073, China; 3 Hunan Provincial Key Laboratory of High Energy Laser Technology, Changsha 410073, China

**Keywords:** Raman fiber amplifiers, transverse mode instability, higher-order mode excitation

## Abstract

Raman fiber lasers (RFLs), which are based on the stimulated Raman scattering effect, generate laser beams and offer distinct advantages such as flexibility in wavelength, low quantum defects, and absence from photo-darkening. However, as the power of the RFLs increases, heat generation emerges as a critical constraint on further power scaling. This escalating thermal load might result in transverse mode instability (TMI), thereby posing a significant challenge to the development of RFLs. In this work, a static model of the TMI effect in a high-power Raman fiber amplifier based on stimulated thermal Rayleigh scattering is established considering higher-order mode excitation. The variations of TMI threshold power with different seed power levels, fundamental mode purities, higher-order mode losses, and fiber lengths are investigated, while a TMI threshold formula with fundamental mode pumping is derived. This work will enrich the theoretical model of TMI and extend its application scope in TMI mitigation strategies, providing guidance for understanding and suppressing TMI in the RFLs.

## 1. Introduction

Raman fiber lasers (RFLs) capitalize on the stimulated Raman scattering (SRS) to achieve laser gain, demonstrating unique potential for the generation of high-power laser output [1,2,3]. The fibers used in RFLs do not necessitate rare-earth (RE) ion doping, thus eliminating the need for specific pump wavelength requirements [4,5,6]. Moreover, with the capability for cascaded operation, RFLs are able to provide laser output at wavelengths that are inaccessible to RE-doped lasers [7,8,9,10,11]. Additionally, RFLs are resistant to challenges such as gain saturation, amplified spontaneous emission, and photon darkening—issues that are critical in the development of high-power RE-doped fiber lasers [12,13]. To date, RFLs have successfully achieved multi-kilowatt laser outputs, with Yb-Raman hybrid gain lasers notably reaching the 10-kW level [14], thereby broadening their applicability in practical scenarios.

At the kilowatt level, Xiao et al. conducted measurements of the beam quality of Yb-Raman hybrid gain lasers across various power levels [15]. Their findings revealed a distinct threshold phenomenon, characterized by a rapid increase in the M^2^ factor from below 1.35 to approximately 1.5. Similarly, certain fiber lasers utilizing pure Raman gain exhibited inferior beam quality, with M^2^ factors degrading to 2.5 [16,17]. These declines in beam quality are likely attributable to mode instability, which may pose a significant challenge to the advancement of high-power RFLs.

In the field of high-power fiber laser technology, the phenomenon of transverse mode instability (TMI), which is a consequence of thermo-optic and nonlinearity effects, has been extensively investigated in ytterbium-doped fiber lasers (YDFLs) [18,19,20,21,22,23,24,25,26,27,28]. However, theoretical and experimental research about TMI of RFLs remains relatively sparse. Distler et al. have explored the effects of varying seed power and fiber lengths on the TMI threshold, albeit within a limited range of parameters [29,30]. Naderi et al. established the first TMI model for Raman gain in Raman fiber amplifiers (RFA), although this model did not take fiber loss into account [31]. Dong subsequently developed a TMI model that incorporates both Raman gain and Yb-Raman hybrid gain in RFA [32], incorporating fiber loss and analyzing the impact of various parameters on the TMI effect, thereby enriching the theoretical model of TMI in RFA. However, these two models mainly focus on passive suppression strategies. In YDFLs, the TMI effect can be suppressed by actively controlling the mode in the fiber. In 2023, Chen et al. proposed a method to mitigate TMI by exciting higher-order modes within the ytterbium-doped fiber amplifier. Their findings indicated that the TMI threshold power increases linearly with the number of similarly excited modes [33]. However, due to the differing gain mechanisms, employing mode-controlled fiber lasers as seed and pump sources in RFA increases the modal count, which could potentially degrade beam quality. Therefore, there is a pressing need to develop a theoretical model that investigates the impact of modal purity on the TMI threshold power in RFA.

In this work, we introduced a higher-order mode (LP_11_) that is incoherent with LP_01_ and simulated the impact of seed and pump purities on the TMI threshold. A static TMI model of RFA, based on stimulated thermal Rayleigh scattering, is established. Additionally, fiber losses and lengths are taken into account in this model. The simulation results indicate that the TMI threshold is influenced by the purities of both the seed and pump, where decreased seed purity and increased pump purity contribute to an increase in TMI threshold. The research findings provide valuable insights for enhancing the TMI threshold in RFA, offering a reference for achieving high-power, high-brightness laser output.

## 2. Theoretical Model

The RFA model uses a few-mode step-index fiber, which allows for the reasonable assumption that both the LP_01_ and LP_11_ modes are present within the system, consistent with most TMI models. It is assumed that the LP_11_ mode from the pump or seed is incoherent with the LP_01_ [34], while the Stokes frequency-shifted LP_11_ is due to quantum noise, and TMI is caused by the interaction between the LP_01_ and the Stokes frequency-shifted LP_11_ [33]. It is important to note that the model does not account for the effects of thermal lensing or the higher-order Raman frequency shift.

Firstly, we establish the power evolution model of RFA. In the case of only first-order Raman frequency shift and only LP_01_ and LP_11_ modes, the power evolution process is described by the following formula [35]:

(1) $$\frac{dP_{P,m}(z)}{dz}=g_{P,m}(z)P_{P,m}(z)-\alpha_{P,m}(z)P_{P,m}(z),m=1,2$$

(2) $$\frac{dP_{S,m}(z)}{dz}=g_{S,m}(z)P_{S,m}(z)-\alpha_{S,m}(z)P_{S,m}(z),m=1,2$$

where *P_P(S)_* is power of pump (signal); *α* is background loss; subscript *m* stands for different modes, where *m* = 1 means LP_01_ mode and *m* = 2 means LP_11_ mode; and *g_m_* is the Raman gain of the *m*-th mode. Equations (1) and (2) are initial value problems, given an initial seed and pump power (*z* = 0), the power at the fiber output (*z* = *L*, *L* is the fiber length) is obtained. Raman gain can be expressed in the following form:

(3) $$g_{P,m}(z)=-\frac{\lambda_{S}}{\lambda_{P}}\sum_{i=1}^{2}\frac{g_{R}(\lambda_{P},\lambda_{S})}{A_{eff(i,m)}(\lambda_{P},\lambda_{S})}P_{S,i}(z),m=1,2$$

(4) $$g_{S,m}(z)=\sum_{i=1}^{2}\frac{g_{R}(\lambda_{P},\lambda_{S})}{A_{eff(i,m)}(\lambda_{P},\lambda_{S})}P_{P,i}(z),m=1,2$$

where *g_R(λp, λs)_* is the Raman gain coefficient of silica, whose value is approximately the order of 10^−14^; *A_eff(i,m)_* is the effective interaction field area between mode *i* and mode *m*; and each mode corresponds to a signal and a pump. In the fiber we calculate that the *A_eff_* between LP_01_ modes (~264 μm^2^) is smaller than that between LP_01_ mode and LP_11_ mode (~424 μm^2^). Therefore, in RFA, the gain of the signal is significantly influenced by the effective interaction field area. *A_eff(i,m)_* is defined by the following [35]:

(5) $$A_{eff(i,m)}=\frac{\int_{0}^{Rclad}\int_{0}^{2\pi }\psi_{P,i}^{2}rd\phi dr\int_{0}^{Rclad}\int_{0}^{2\pi }\psi_{S,m}^{2}rd\phi dr}{\int_{0}^{Rclad}\int_{0}^{2\pi }\psi_{P,i}^{2}\psi_{S,m}^{2}rd\phi dr},i=1,2\;\&\;m=1,2$$

where *ψ* is the normalized mode distribution; *r* and *φ* are radial coordinates and angular coordinates, respectively; and *R_clad_* is the radius of the fiber cladding.

According to the RFA model described above, we can obtain the gain amplification process for both the fundamental mode and higher-order modes at the same frequency. Although the fundamental mode and higher-order modes at the same frequency can form a static long-period refractive index grating, the static grating does not cause dynamic mode coupling. Only the dynamic refractive index grating formed between the fundamental mode and higher-order modes with Stokes frequency shift can lead to dynamic mode coupling, resulting in the TMI phenomenon.

According to the thermo-optic effect, the dynamic thermally-induced refractive index grating generated by the inter-mode interference field between the LP_01_ mode and LP_11_ mode with the frequency shift Ω can be expressed as follows:

(6) $$\Delta n(r,\phi ,z,t)=k_{T}T(r,\phi ,z,t)$$

(7) $$\rho C\frac{\partial T\left(r,\phi ,z,t\right)}{\partial t}-\kappa \nabla^{2}T\left(r,\phi ,z,t\right)=Q\left(r,\phi ,z,t\right)$$

where *k_T_* is the thermo-optic coefficient; *T* is the temperature distribution; *κ*, *ρ*, and *C* are the thermal conductivity, density, and specific heat of silica, respectively; and *Q* is the heat density distribution generated by the interference field, which can be expressed as follows:

(8) $$Q\left(r,\phi ,z,t\right)=q_{D}g_{R}I_{P}(r,\phi ,z)A_{1}(z,t)A_{Stokes}^{*}(z,t)\psi_{s,1}(r,\phi )\psi_{s,2}(r,\phi )e^{i(\Delta \beta z-\Omega t)}$$

where $q_{D}=\lambda_{S}/\lambda_{P}-1$ is the quantum defect of Raman conversion; *A*_1_ is the amplitude of LP_01_ mode; *A_Stokes_* is the amplitude of LP_11_ mode with the frequency shift Ω; *I_P_* is the light intensity of pump; and Δ*β* is the difference of propagation constants between LP_01_ mode and LP_11_ mode. Using Green’s function method, the thermally induced refractive index change can be expressed as follows:

(9) $$\begin{array}{ll} \Delta n\left(r,\phi ,z,t\right) & =k_{T}\sum_{v=0}\sum_{l=1}J_{v}(k_{vl}r)\\ & \frac{\int_{0}^{2\pi }\int_{0}^{Rclad}q_{D}g_{R}I_{P}(r^{\prime },\phi^{\prime },z)A_{1}(z,t)A_{Stokes}^{*}(z,t)\psi_{s,1}(r^{\prime },\phi^{\prime })\psi_{s,2}(r^{\prime },\phi^{\prime })\left(r^{\prime },\phi^{\prime },z,t\right)J_{v}(k_{vl}r^{\prime })\cos[v(\phi -\phi^{\prime })]r^{\prime }dr^{\prime }d\phi^{\prime }}{(\kappa k_{vl}^{2}-i\Omega \rho C)\int_{0}^{2\pi }\int_{0}^{Rclad}J_{v}^{2}(k_{vl}r)\cos^{2}(v\phi )rdrd\phi }e^{i(\Delta \beta z-\Omega t)} \end{array}$$

where *k_vl_* is the *l*-th positive root of the equation $J_{v}(k_{vl}r_{clad})=0$.

Based on the nonlinear propagation equation, $\nabla^{2}E-{\left(\frac{n}{c}\right)}^{2}\frac{\partial^{2}E}{\partial t^{2}}=\frac{1}{\varepsilon_{0}c^{2}}\frac{\partial^{2}P^{NL}}{\partial t^{2}}$, the nonlinear polarization intensity, $P^{NL}\approx 2n\varepsilon_{0}E\Delta n$, the inter-mode coupling equations between the LP_01_ mode and LP_11_ mode with Stokes frequency shift can be rewritten as follows:

(10) $$\frac{\partial P_{S,1}}{\partial z}=(g_{S,1}-\alpha_{S,1})P_{S,1}(z)-g_{S,1}\chi P_{S,1}(z)P_{Stokes}(z)$$

(11) $$\frac{\partial P_{Stokes}}{\partial z}=(g_{S,2}-\alpha_{S,2})P_{Stokes}(z)-g_{S,1}\chi P_{S,1}(z)P_{Stokes}(z)$$

where *P_Stokes_* is the LP_11_ mode power with the Stokes frequency shift. The nonlinear coupling coefficient *χ* is as follows:

(12) χ=2k0Re∫02π∫0claddingiΔnA1∗(z,t)AStokes(z,t)ψ1(r,ϕ)ψ2(r,ϕ)e−i(Δβz−Ωt)rdrdgS,1PS,1(z,t)PStokes(z,t)

where *k*_0_ is the wave number and Re denotes taking the real part.

The total gain of mode LP_11_ mode with the frequency shift Ω is shown below:

(13) $$G(z,\Omega )=g_{1}(z)\chi (\Omega )P_{1}(z)+g_{S,2}(z)-\alpha_{S,2}(z)$$

Thus, the power of Stokes frequency-shifted LP_11_ can be expressed as follows:

(14) $$P_{Stokes}(z,\Omega )=P_{Stokes}(0)\cdot \exp[\int_{0}^{z}G(z,\Omega )dz]$$

In the simulation, the power ratio of the Stokes frequency-shifted LP_11_ mode to signal LP_01_, denoted as $\xi \left(z\right)=P_{Stokes}\left(z\right)/P_{S,1}\left(z\right)$ to characterize the TMI effect. When the ratio *ξ*(*L*) equal to 0.05, defined TMI occurs, the total signal output power is defined as TMI Threshold.

## 3. Results and Discussion

**Figure 1:**
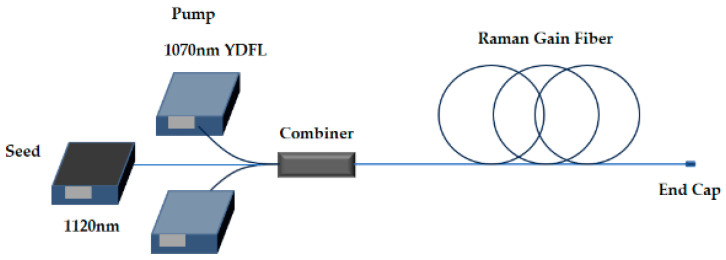
Schematic diagram of the RFA structure.

The purity is defined by the fundamental mode content, specifically the ratio of the power of LP_01_ to the total signal or pump power. It should be noted that the purity, whether pumped or seeded, ranges from 0.6 to 1. The simulation structure is shown in Figure 1, where the pump and signal are injected into the Raman gain fiber through the combiner and output through the end cap. The initial Stokes frequency-shifted LP_11_ power is set to 10^−9^ of the initial LP_01_ power, which is consistent with ref. [32]. Other parameters in the model are listed in Table 1.

### 3.1. The TMI Threshold Changes with the Seed When the Pump Purity Is 1.0

In this section, it is assumed that the purity of the pump is 1.0, which corresponds to the case of pure LP_01_ mode pumping. From Equations (9) and (12), it can be observed that when the pump exists only LP_01_, the nonlinear coupling coefficient can be approximated as a constant. Therefore, regardless of loss, the output power of the signal LP_01_ can be expressed as follows:

(15) $$P_{S,1}(L)-\frac{M}{\chi }\ln P_{S,1}(L)=\frac{\ln\left(\xi (L)/\xi (0)\right)}{\chi }+P_{S,1}(0)-\frac{M}{\chi }\ln P_{S,1}(0)$$

(16) $$M=\left(1-\frac{A_{eff(1,1)}}{A_{eff(1,2)}}\right)$$

It can be seen from Equation (15) that when TMI occurs, if the power of LP_01_ in the seed is fixed, the power of LP_01_ at output is also fixed. However, the output power of the LP_01_ does not vary linearly with the change in the LP_01_ power in seed.

According to Equation (2), the relationship between the signal LP_11_ output power and the power of the LP_01_ in seed is given by the following:

(17) $$P_{S,2}(L)=P_{S,1}(0)e^{-M}\cdot \left[\frac{1}{purity}-1\right]$$

**Figure 2:**
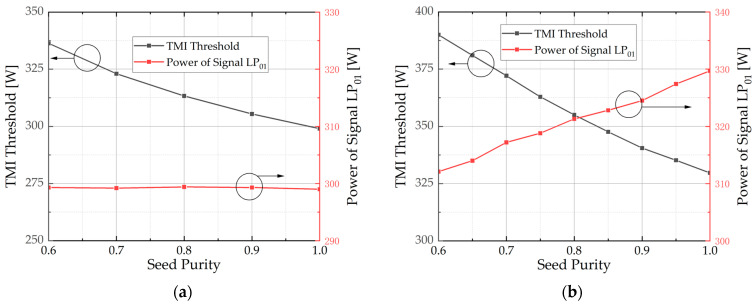
The change in TMI threshold and output signal LP_01_ power with varying seed purities. (**a**) When the LP_01_ power in seed is fixed at 5 W; (**b**) When the seed power is fixed at 50 W.

The purity here refers to the seed purity. Based on Equations (15)–(17), two conclusions can be drawn: Firstly, when the power of the LP_01_ in the seed is fixed, the output signal LP_01_ power remains constant. As the seed purity decreases, the incoherent LP_11_ power increases, resulting in an increase in the TMI threshold. Secondly, with the seed power held constant, an increase in seed purity leads to a reduction in the incoherent LP_11_ power of the output signal. The variation of the output LP_01_ power, however, does not exhibit a monotonic trend. Therefore, it is not possible to directly summarize the change in the TMI threshold. Simulations were performed to address these two scenarios, and the findings are presented in Figure 2. The fiber length used in the simulation is 20 m, and the fiber loss is ignored. Other parameters are consistent with Table 1.

Figure 2a shows the change of TMI threshold and output signal LP_01_ power with different seed purities when LP_01_ power in the seed is 5 W. The LP_01_ power in the output signal is maintained at 299 W, while the TMI threshold increases as the seed purity decreases. When the seed purity is 0.6, the TMI threshold increases to 336 W. The simulation results demonstrate that a decrease in seed purity leads to an increase in the TMI threshold. This is because the occurrence of TMI is related to the power of LP_01_. Consequently, with equal LP_01_ power, an increase in the LP_11_ power within the seed leads to an increased TMI threshold.

The change in TMI threshold and output signal LP_01_ power with varying seed purities is illustrated in Figure 2b, while keeping the seed power fixed at 50 W. As the seed purity increases, both the TMI threshold and LP11 power of the output signal exhibit a monotonic trend. The TMI threshold was 330 W when the seed purity was 1.0. However, by reducing the seed purity to 0.6, the TMI threshold increases to 392 W.

**Figure 3:**
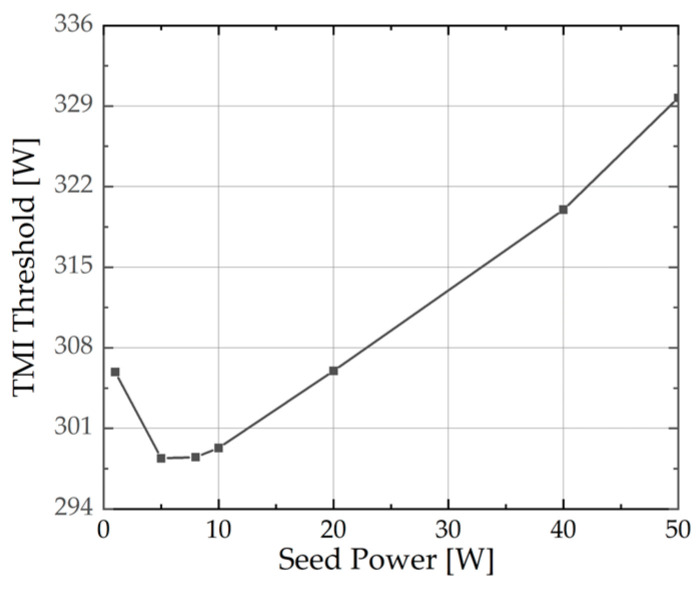
The TMI threshold under different seed power.

When the seed purity is 1.0, the TMI threshold is higher at 50 W seed power, compared to the case at 5 W. To further investigate the impact of seed power on the TMI threshold, simulations were conducted with a seed purity of 1 at various seed power levels, while all other conditions remained constant. The results are depicted in Figure 3. As illustrated, the TMI threshold first decreases and then increases, reaching its minimum at a seed power of 8 W. By comparing the trends of the LP_01_ power in Figure 3 and Figure 2b, it is observed that if the LP_01_ power in the seed is equal, its output power is also equal. The simulation results are in accordance with our discussions regarding the Equations (15)–(17). The monotonic increase in LP_01_ power in Figure 2b may be attributed to its power variation range of 30 to 50 W. Within this range, as depicted in Figure 3, the power exhibits a consistent upward trend.

The TMI threshold of 50 W seed power reaches its maximum when the seed purity is 1.0. Therefore, in Section 3.2, the seed power of 50 W was used to study the effect of different pump purities on the TMI threshold in the case of seed purity equals to 1.0.

### 3.2. The TMI Threshold Changes with the Pump Purity When the Seed Purity Is 1.0

In Section 3.1, the impact of seed purity on the TMI threshold in RFA is discussed under the assumption of an ideal pumping purity of 1.0. Under core-pumped conditions, the presence of higher-order modes in the pumping can result in an enhancement of the Raman gain for the higher-order mode of the signal. The influence of pump purity on the TMI threshold will be discussed in this section.

**Figure 4:**
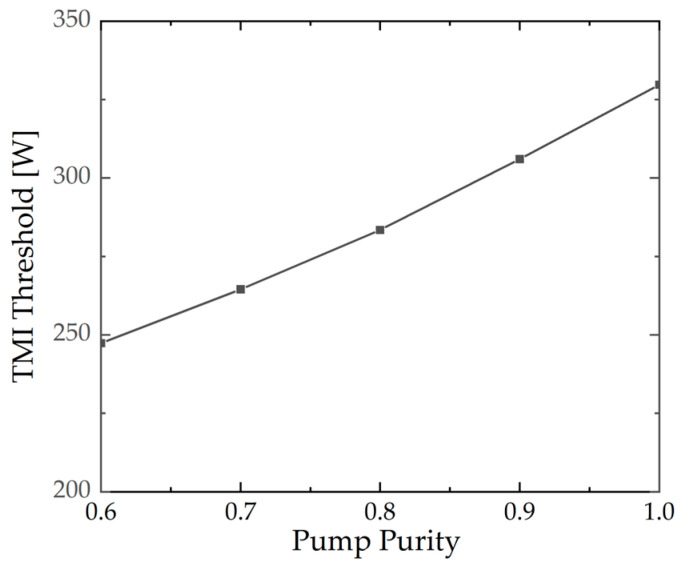
The TMI threshold under different pump purities.

Figure 4 shows the TMI threshold for different pump purities. The seed power is maintained at 50 W, and the seed purity is set to 1.0. Other parameters are consistent with Section 3.1. It is evident that the relationship between TMI threshold and pump purity is opposite to that between TMI threshold and seed purity. Specifically, the TMI threshold is observed to increase with pump purity; it increases from 247.3 W at a pump purity of 0.6 to 329.7 W at a pump purity of 1.0. In this theoretical model, the power of both the LP_01_ and the LP_11_ of the pump can be coupled to the Stokes frequency-shifted LP_11_ mode through Raman conversion. The *A_eff_* between the LP_11_ modes is smaller than that between the LP_11_ and LP_01_. Consequently, at lower pump purity, the gain of the Stokes frequency-shifted LP_11_ is higher, resulting in a lower TMI threshold. Therefore, maintaining the high beam quality of the pump in experiments can lead to a higher TMI threshold.

**Figure 5:**
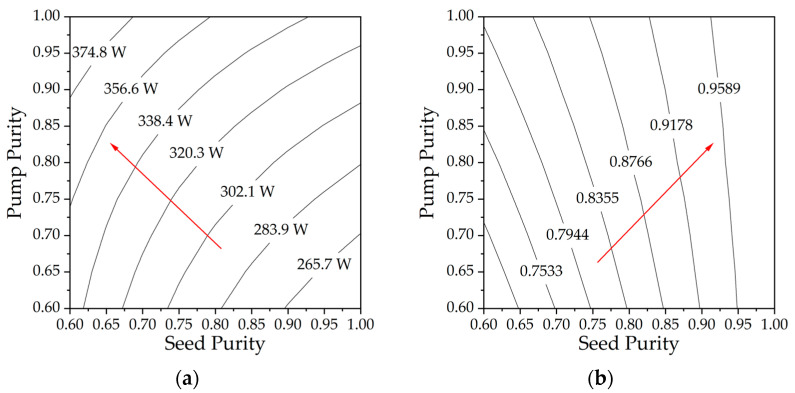
(**a**) The TMI threshold and (**b**) the LP_01_ content varies with different seed and pump purities.

According to Section 3.1 and the conclusions in this section, we can tentatively synthesize the impact of mode content on the TMI threshold. Combined with data corresponding to various seed and pump purities, utilizing a seed power of 50 W, as shown in Figure 5, while other parameters held constant as shown in Table 1. Figure 5a illustrates the variations in TMI threshold for different seed and pump purities. It is observed that as the seed purity decreases and the pump purity increases, there is a corresponding increase in the TMI threshold. Figure 5b shows the ratio of signal LP_01_ power to the total signal output power for different seed and pump purities. It can be observed that a higher purity of both the seed and pump leads to an increase in LP_01_ content. Therefore, in RFA, the purity of the pump should ideally be maintained as high as possible, while the purity of the seed should be adjusted according to the specific application scenarios to balance the high TMI threshold and the high LP_01_ content.

### 3.3. Influence of Fiber Length and Loss on TMI Threshold

The previous sections systematically analyzed the influence of mode excitation on the TMI threshold in RFA. This section will focus on strategies to increase the TMI threshold. Theoretically, the attenuation of higher-order modes significantly influences the TMI threshold. Consequently, Section 3.3.1 will investigate the impact of varying LP_11_ loss on the TMI threshold during the transmission process. Further, Section 3.3.2 will consider the LP_11_ loss to study the impact of different fiber lengths on the TMI threshold. Considering the results of the previous simulation, the seed purity is set to 0.6, the pump purity is set to 0.9, and the seed power is set to 50 W.

#### 3.3.1. Influence of LP_11_ Mode Loss on TMI Threshold

In this subsection, the TMI threshold change under different LP_11_ losses is studied. For the convenience of analysis, the loss of the LP_11_ of both pump and seed (both are equal) will be changed while setting the attenuation of the LP_01_ to 0 dB/m, with all other parameters remaining unchanged as detailed in Table 1.

**Figure 6:**
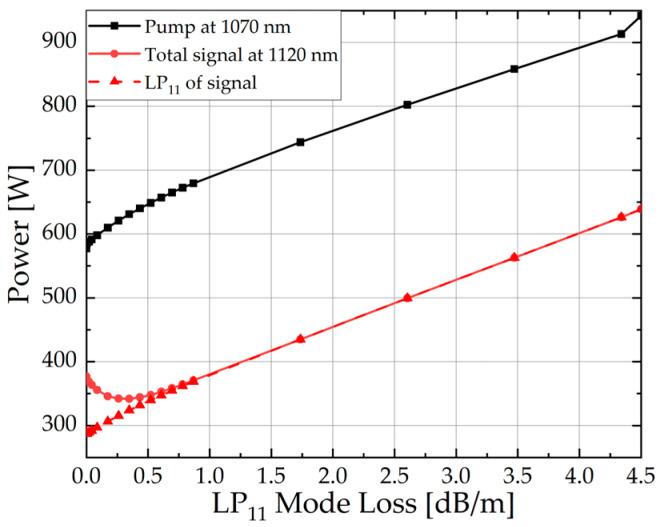
The pump power, total signal power, and LP_01_ power of the signal under different LP_11_ mode losses when TMI occurs.

As shown in Figure 6, as the LP_11_ loss increases, the output signal power of LP_01_ increases linearly. In the case of low loss, the total output power of the signal decreases because the growth of the LP_01_ mode cannot compensate for the loss of the LP_11_ mode. This further affects the change of the TMI threshold.

**Figure 7:**
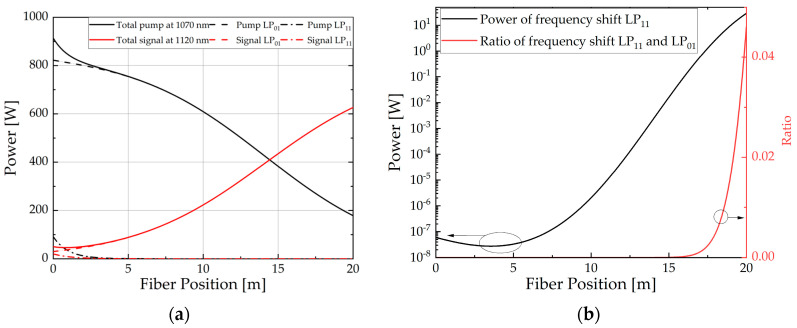
(**a**) Power evolution in RFA; (**b**) Power evolution and proportion of Stokes frequency-shifted LP_11_ mode at different positions along the fiber under a LP_11_ mode loss of 4.34 dB/m.

The power evolution of Raman conversion is depicted in Figure 7a for the LP_11_ mode loss of 4.34 dB/m. Concurrently, the evolution and distribution of power for the Stokes frequency-shifted LP_11_ mode are illustrated in Figure 7b. As shown in Figure 7b, the power of Stokes frequency-shifted LP_11_ decreases initially due to the significant loss, which is the primary reason for the increase in the TMI threshold. According to Figure 7a, the signal output power is 626.3 W at the high LP_11_ mode loss. The purity of the fundamental mode approaches 1.0, the injection pump power reaches 913.4 W, and the Raman conversion efficiency is 63.1%. Therefore, during specific experiments, increasing the LP_11_ mode loss by bending the fiber or using functional passive fiber components [36] will increase the TMI threshold and increase the output signal fundamental mode content.

#### 3.3.2. Influence of Fiber Length on TMI Threshold

**Figure 8:**
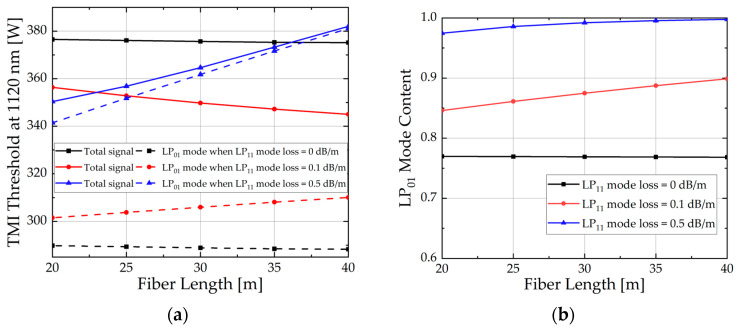
(**a**) Total output signal power and LP_01_ power of different fiber lengths; (**b**) LP_01_ mode purity at TMI thresholds of different fiber lengths.

In this subsection, the influence of fiber length on the TMI threshold is studied when the LP_11_ mode loss is 0, 0.1, and 0.5 dB/m, respectively. The LP_01_ loss is ignored, and other parameters remain consistent with Table 1. The results are shown in Figure 8.

As observed in Figure 8a, when there is no loss in the fiber, the TMI threshold appears to be largely independent of the fiber length. However, when considering LP_11_ loss, a greater loss results in a more rapid attenuation of the Stokes frequency-shifted LP_11_. This delays the emergence of the TMI effect, thereby improving the power of the LP_01_.

## 4. Conclusions

Considering mode excitation, a static model of TMI in RFA is established based on stimulated thermal Rayleigh scattering and power coupling theory in this work. Higher-order mode at the same frequency as fundamental mode in RFA is introduced, and the effect of different seed and pump purities on the TMI threshold is simulated. The simulation results indicate that the TMI threshold decreases with the increase in seed purity. And it first decreases and then increases with the increase in seed power. However, reducing the seed purity will decrease the output power of the LP_01_, which needs a trade-off in practical applications. Contrary to the effect of seed purity, an increase in pump purity is directly associated with an increased TMI threshold, implying that a high-brightness pump source is essential. The influence of LP_11_ losses and fiber lengths on the TMI threshold of RFA is essentially the influence on the frequency-shifted LP_11_ power. An increase in the loss of frequency-shifted LP_11_ corresponds to a reduction in the gain as described by Equation (13), consequently leading to an increased TMI threshold. The research findings provide new insights into enhancing the TMI threshold in RFA, offering a reference for achieving high-power, high-brightness laser output.

## Figures and Tables

**Table 1:** The main simulation parameters of Raman fiber amplifiers.

Parameter	Value	Parameter	Value
$\lambda_{P}$	1070 nm	κ	1.38 W/(m·K)
$\lambda_{S}$	1120 nm	$k_{T}$	1.2 × 10^−5^ K^−1^
$n_{core}$	1.4662	$g_{R}$	7.3 × 10^−14^ m/W
$n_{clad}$	1.465	ρC	1.63 × 10^6^ J/(K·m^3^)
$2r_{core}$	20 μm	$2r_{clad}$	130 μm

## Data Availability

The data that support the findings of this study are available from the authors upon reasonable request.
